# Understanding of Statistical Terms Routinely Used in Meta-Analyses: An International Survey among Researchers

**DOI:** 10.1371/journal.pone.0047229

**Published:** 2013-01-11

**Authors:** Michael N. Mavros, Vangelis G. Alexiou, Konstantinos Z. Vardakas, Matthew E. Falagas

**Affiliations:** 1 Alfa Institute of Biomedical Sciences (AIBS), Athens, Greece; 2 Department of Surgery, University Hospitals of Leicester, Leicester, United Kingdom; 3 Department of Medicine, Mitera Hospital, Hygeia Group, Athens, Greece; 4 Department of Medicine, Tufts University School of Medicine, Boston, Massachusetts, United States of America; Universidad Peruana Cayetano Heredia, Peru

## Abstract

**Objective:**

Biomedical literature is increasingly enriched with literature reviews and meta-analyses. We sought to assess the understanding of statistical terms routinely used in such studies, among researchers.

**Methods:**

An online survey posing 4 clinically-oriented multiple-choice questions was conducted in an international sample of randomly selected corresponding authors of articles indexed by PubMed.

**Results:**

A total of 315 unique complete forms were analyzed (participation rate 39.4%), mostly from Europe (48%), North America (31%), and Asia/Pacific (17%). Only 10.5% of the participants answered correctly all 4 “interpretation” questions while 9.2% answered all questions incorrectly. Regarding each question, 51.1%, 71.4%, and 40.6% of the participants correctly interpreted statistical significance of a given odds ratio, risk ratio, and weighted mean difference with 95% confidence intervals respectively, while 43.5% correctly replied that no statistical model can adjust for clinical heterogeneity. Clinicians had more correct answers than non-clinicians (mean score ± standard deviation: 2.27±1.06 *versus* 1.83±1.14, p<0.001); among clinicians, there was a trend towards a higher score in medical specialists (2.37±1.07 *versus* 2.04±1.04, p = 0.06) and a lower score in clinical laboratory specialists (1.7±0.95 *versus* 2.3±1.06, p = 0.08). No association was observed between the respondents' region or questionnaire completion time and participants' score.

**Conclusion:**

A considerable proportion of researchers, randomly selected from a diverse international sample of biomedical scientists, misinterpreted statistical terms commonly reported in meta-analyses. Authors could be prompted to explicitly interpret their findings to prevent misunderstandings and readers are encouraged to keep up with basic biostatistics.

## Introduction

Literature reviews, including systematic reviews and meta-analyses, are critical components of evidence-based medicine. Such studies are commonly regarded as valuable sources of evidence and influence both clinical practice and public health policy [1], [2]. Following the expansion of published biomedical original research, the publication of literature reviews has also greatly increased [3]. Systematic reviews and meta-analyses are expected to accumulate and synthesize the total body of evidence regarding a topic and present it in a way that is comprehensible to busy health practitioners.

Statistical terms commonly used in meta-analyses, but also original research studies, include effect estimate measures such as the odds ratio (OR), risk ratio (RR), and weighted mean difference (WMD). Another important component of evidence synthesis studies is heterogeneity, which can be classified as clinical or statistical heterogeneity. Previous studies have implied a suboptimal understanding of such statistical terms among readers and/or researchers, but no study to our knowledge has assessed the understanding of plain effect estimates, provided in a commonly-encountered, clinical context. In this regard, we sought to investigate the current level of comprehension of statistical terms commonly used in meta-analyses.

## Methods

### Survey design and participants

An on-line survey was conducted from December 2011 to January 2012, based on the methodology of electronic surveys previously published [4]–[7]. Briefly, we selected a random sample of PubMed unique identifiers (PMID) between 10,000,000 and 22,000,000 (mostly referring to articles published during the last 15 years), using a random number generator [8]. We established communication with the corresponding authors who had an e-mail address available at the indexed affiliation and asked them to voluntarily complete an open, web-based questionnaire [9]; this study was not announced or advertised, and access to the questionnaire by non-invited individuals was unlikely. By using this approach, we tried to survey a random, representative, and diverse international sample of researchers. In case of duplicate responses (posted from the same IP address within 24 hours), only the first response was analyzed.

Participants were informed about the aims of the study, the length of time of the survey, and the primary investigator (MEF). The questionnaire was a structured, web-based, multiple-choice form, comprising of 5 single-answer questions. Four mandatory questions evaluated the understanding of simple statistical terms commonly used in meta-analyses (OR, RR, WMD, and heterogeneity), in a clinical context, and the last, optional question, inquired the specialty of the respondent (Table 1). We also recorded the questionnaire completion time and the participants' country of origin as derived by their Internet Protocol (IP) address; no other personal information was collected. Answers were submitted electronically to ensure anonymity of the participants. The survey and study protocol were approved by the Ethics Committee of the Alfa Institute of Biomedical Sciences (AIBS), Athens, Greece. Informed consent of the participants was implied by the completion and electronic submission of the questionnaire. The study has been described in concordance with the CHERRIES (Checklist for Reporting Results of Internet E-Surveys) guidelines [10].

**Table 1:** Our questionnaire.

1) A meta-analysis of randomized controlled trials (RCTs) compared a new drug *versus* an old drug for the cure of a defined infection; the pooled odds ratio (OR) was 0.61 [95% confidence intervals (CI): 0.41 to 0.91]. According to this meta-analysis:a) The new drug is more effective than the old one.**b) The new drug is less effective than the old one.**c) The new and the old drugs are equally effective.d) I am not sure.
2) A meta-analysis of RCTs compared a new drug *versus* an old drug with regard to the incidence of nephrotoxicity post-treatment; the pooled risk ratio (relative risk, RR) was 1.05 (95% CI: 0.51 to 2.19). According to this meta-analysis:a) The new drug is more nephrotoxic than the old one.b) The new drug is less nephrotoxic than the old one.**c) The new and the old drugs are equally nephrotoxic.**d) I am not sure.
3) A meta-analysis of RCTs compared a new drug *versus* an old drug with regard to the patients' length of hospital stay (LOS); the weighted mean difference (WMD) was 2.63 (95% CI: 0.22 to 5.04). According to this meta-analysis:**a) Patients receiving the new drug had longer LOS.**b) Patients receiving the new drug had shorter LOS.c) Patients in both groups had a similar LOS.d) I am not sure.
4) A meta-analysis was conducted, pooling studies with clinical heterogeneity but without substantial statistical heterogeneity (p>0.1, I^2^ = 30%). Which of the following statistical models would be appropriate for this meta-analysis?a) The fixed effect model.b) The random effects model.c) Another model.**d) No statistical model can adjust for clinical heterogeneity.**
5) Your specialty is:a) Medical (including psychiatry)b) Surgical (including anesthesiology)c) Clinical laboratory (including radiology)d) None of the above

The correct answers (when applicable) are in **bold**.

### Data analysis and statistical methods

Respondents' answers were pooled and graphically presented. A score was calculated for each participant, representing the number of correct answers (1 point was awarded for each correct answer). Univariate comparisons were performed to examine the potential effect of respondents' specialty, region, and questionnaire completion time on their score. We used Pearson correlation, Student's t-test, and analysis of variance tests for normally distributed variables, and Spearman correlation, Mann-Whitney, and Kruskal-Wallis (for non-parametrically distributed variables) tests, as appropriate. The normality of the distribution of the variables was assessed with the Wilk-Shapiro test. All analyses were performed with STATA 11.2 (Stata Corp., College Station, TX, USA) statistical software package. A p<0.05 was considered to denote statistical significance.

## Results

The online questionnaire was accessed 800 times and after exclusion of 1 duplicate report, a total of 315 complete forms were analyzed (participation rate 39.4%). The median questionnaire completion time was 202 seconds (interquartile range: 143 to 362 seconds). Most participants completed the questionnaire from Europe (151/315, 48%) and North America (99, 31%), and fewer from Asia/Pacific (52, 17%) and Central & South America or Africa (13, 4%). Most of the participating physicians (n = 169; 16/315 respondents did not provide relevant data) had a medical specialty (69%, 116/169; including psychiatry), while 25% (43/169) had a surgical specialty (including anesthesiology) and few (6%, 10/169) had a clinical laboratory specialty (including radiology). 130 respondents were non-clinicians (non-physicians or physicians without specialty).

**Figure 1:**
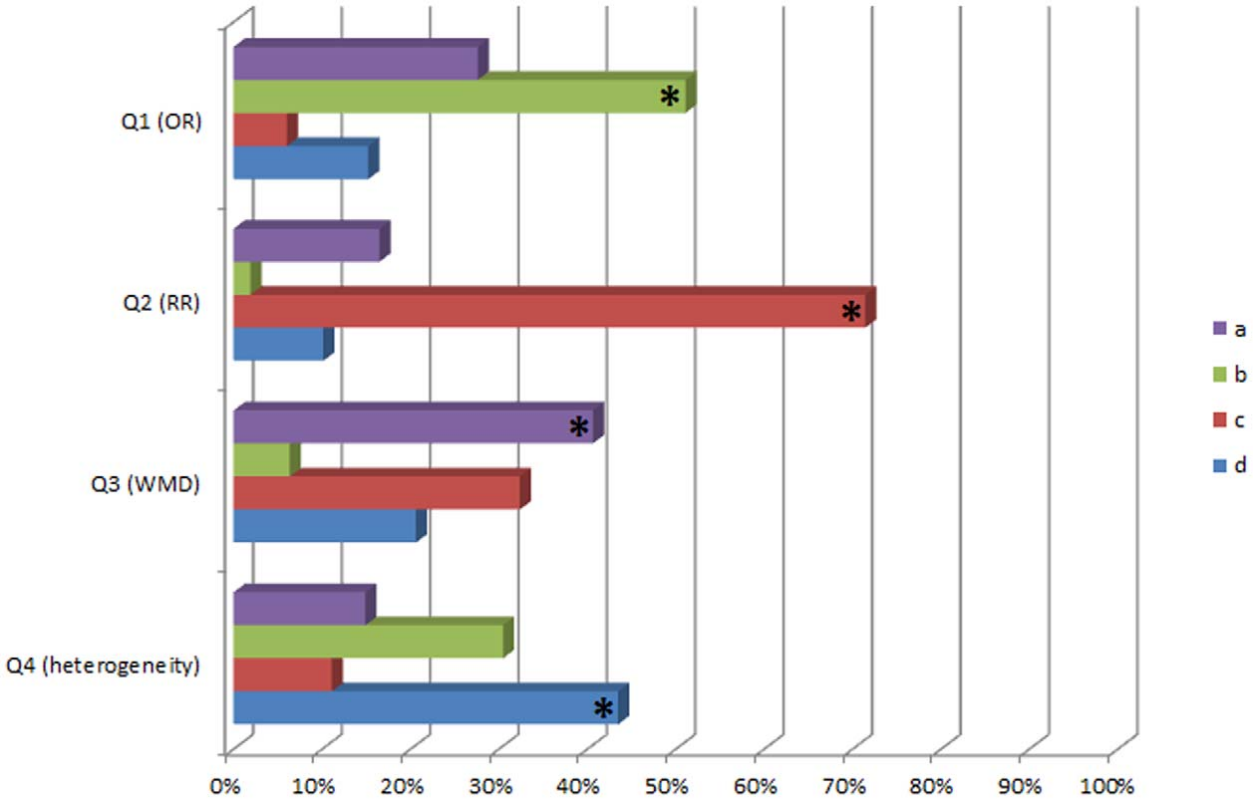
The responses of the participating researchers to each question. Correct answers are marked with an asterisk; the questionnaire is presented in Table 1. [Q = Question; OR = Odds Ratio; RR = Risk Ratio; WMD = Weighted Mean Difference].

Responses to our questions are presented in Figure 1. Overall, almost half of the ‘meta-analysis interpretation’ questions had been answered correctly (51.7%, 651/1260). Thirty-three (10.5%) respondents answered correctly all 4 questions, while 29 (9.2%) answered incorrectly all 4 questions. Almost one third of the respondents (111, 35.2%) answered at least 3 of 4 questions correctly. Regarding each question (Figure 1), 51.1% (161/315), 71.4% (225/315), and 40.6% (128/315) of the participants correctly interpreted statistical significance (or lack of statistical significance) for a given OR, RR, and WMD estimate (with 95% confidence intervals), respectively. Less than half (43.5%, 137/315) of the participants correctly responded that no statistical model can adjust for clinical heterogeneity in meta-analyses.

**Figure 2:**
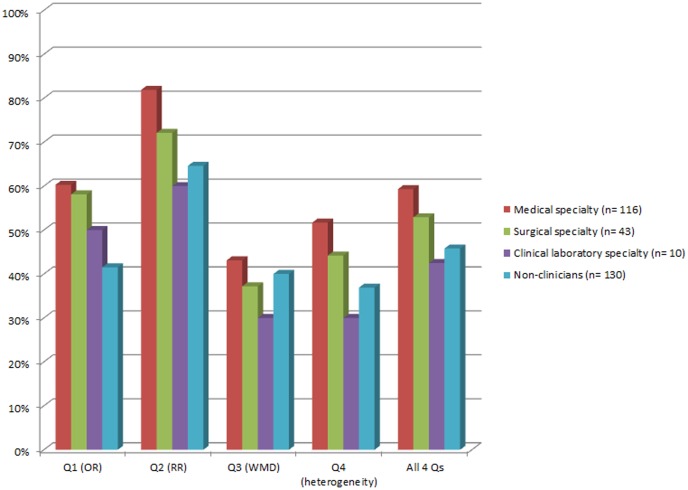
Percentage of correct responses to each question, stratified by specialty. Clinicians had more correct answers than non-clinicians (mean score ± standard deviation: 2.27±1.06 *versus* 1.83±1.14, p<0.001). [Q = Question; OR = Odds Ratio; RR = Risk Ratio; WMD = Weighted Mean Difference].

The percentages of correct responses to each question among the respondents' groups are presented in Figure 2. Clinicians had a higher score than non-clinicians (mean score ± standard deviation: 2.27±1.06 *versus* 1.83±1.14, p<0.001). Among clinicians, there was a trend towards a higher score in medical specialists *versus* the others (2.37±1.07 *versus* 2.04±1.04, p = 0.06) and towards a lower score in clinical laboratory specialists *versus* the others (1.7±0.95 *versus* 2.3±1.06, p = 0.08). No statistically significant difference was observed between surgeons *versus* other specialists (2.12±1.1 *versus* 2.32±1.05, p = 0.28). There was no difference in the score with regard to the respondents' region (Europe 2.12±1.09, North America 2.08±1.1, Asia/Pacific 1.98±1.24, and Central & South America and Africa 1.85±0.99; p = 0.62). There was no correlation between questionnaire completion time and participants' score (p = 0.25).

## Discussion

The main finding of our survey is that, even among researchers, there is incomplete understanding of statistical terms commonly reported in meta-analyses. This finding was more pronounced in non-clinicians; among clinicians, those with a medical specialty tended to have a slightly better understanding of statistical terms than the others. Although the questions were clinically oriented and commonly encountered in the biomedical literature, overall, almost half (48.3%) were answered incorrectly; 10.5% of the respondents answered correctly all questions, while 9.2% answered all questions incorrectly.

Few studies have addressed the level of comprehension of commonly used statistical terms among the providers and the recipients of biomedical research (authors and readers). Previous studies noted an incomplete understanding of the difference between odds ratio and risk ratio, in terms of both calculation [6] and interpretation [11], [12], even among researchers [6]. Others reported that the use of relative (i.e. OR, RR) instead of absolute (i.e. number needed to treat) estimate measures led to an overestimation of the effect by the readers [13], [14]. Although limited published data have suggested an incomplete understanding of basic biostatistics, i.e. the difference between odds ratio and risk ratio, this is the first study to the best of our knowledge to assess the interpretation of plainly given effect estimates. Surprisingly, almost half of the given estimates (OR, RR, WMD) were misinterpreted by corresponding authors of articles indexed in PubMed.

Our findings suggest a better understanding of the tested statistical terms among clinicians, compared with non-clinicians. Clinicians with a medical specialty tended to score higher than the rest. Interestingly, the groups that tended to score higher were the ones that were mostly represented in our analysis (169 clinicians *versus* 130 non-clinicians, 116 medical specialists *versus* 53 surgical/clinical laboratory specialists). This may indicate a higher degree of understanding among clinicians who publish more (as derived from our analysis). Of note, in the United States, medical graduates entering a surgical specialty have higher medical licensing examination scores than their medical and clinical laboratory counterparts [15]; no such trend was observed in our sample.

Our study has significant implications. It has already been argued that a large part of published biomedical research is inaccurate [16], [17]. Adding the fact that commonly used statistical terms are misinterpreted by the readers, the conclusion could be particularly troublesome. Hopefully, most of the misunderstandings are resolved through the own article's interpretation of results. In this regard, it is of paramount importance that the readers have the ability to self-interpret published research findings, especially since some medical journals currently ask the authors to present “appropriate indicators of measurement error or uncertainty (such as confidence intervals) [and] avoid relying solely on statistical hypothesis testing, such as the use of *P* values” [18].

Although through this study we cannot identify the source of the problem, nor suggest a practical solution, the first step in the problem solving process remains the definition and identification of the problem. Our study also serves as a call for careful consideration of published research by journal editors, article authors, and readers. At the end of the day, in this era of rapidly evolving evidence-based medicine, physicians would rather be able to properly interpret current research findings than memorize a large amount of potentially outdated information.

One might argue that our findings should not be generalized to the majority of physicians or biomedical scientists. However, the participants in our survey were corresponding authors of articles indexed by PubMed, who in general are expected to be more statistically knowledgeable than ordinary readers; in addition, the participants represented a random, international sample of scientists and physicians of various specialties. Another potential explanation for our findings would be that the participants did not pay adequate attention to the questions; this is unlikely, considering that those not interested in our survey would not complete and submit it (only complete responses were assessed), and that the median completion time was around 3 minutes (for 4 “interpretation” questions); in this regard, it should be acknowledged that the participation rate was relatively low (39.4%), which is not unusual for this type of research. Last, our study suffers the inherent limitations of online surveys, including self-selection bias and concerns on the accuracy and reproducibility of the responses [19], [20]. In this regard, specific details as to how many publications were screened, how many emails were sent, and how many email addresses were invalid were not available; therefore, we could not exclude the possibility that some regions were under-represented due to self-selection bias. However, the representation of each region in our survey was similar with the global relative biomedical research productivity [21]–[24].

In conclusion, a large proportion of biomedical researchers misinterpreted simple effect estimates commonly used in meta-analyses. Journal editors and article authors may embrace a more comprehensive interpretation of each study's findings, while readers are encouraged to keep up with basic biostatistics.
